# A Water-Soluble Polyacid Polymer Based on Hydrophilic Metal–Organic Frameworks Using Amphoteric Carboxylic Acid Ligands as Linkers for Hydroxycamptothecin Loading and Release In Vitro

**DOI:** 10.3390/nano11112854

**Published:** 2021-10-26

**Authors:** Yuqiong Shi, Wei Liu, Xiangrong Wu, Jinhua Zhu, Danyang Zhou, Xiuhua Liu

**Affiliations:** Henan International Joint Laboratory of Medicinal Plants Utilization, College of Chemistry and Chemical Engineering, Henan University, Kaifeng 475004, China; sq13027537872@163.com (Y.S.); 104752190079@henu.edu.cn (W.L.); 16639791569@163.com (X.W.); aries1075@163.com (D.Z.)

**Keywords:** polyacid polymer, metal organic frameworks, HCPT delivery

## Abstract

The poor water solubility and severe side effects of hydroxycamptothecin (HCPT) limit its clinical application; therefore, it is necessary to synthesize applicable nanodrug carriers with good solubility to expand the applications of HCPT. In this study, a hydrophilic metal–organic framework (MOF) with amphoteric carboxylic acid ligands as linkers was first synthesized and characterized. Then, water-soluble acrylamide and methacrylic acid were applied as monomers to prepare a water-soluble polyacid polymer MOF@P, which had a solubility of 370 μg/mL. The effects of the MOF@P material on the HCPT loading and solubility were investigated. The results showed that the polymer material could improve the HCPT solubility in water. Moreover, the in vitro release study indicated that the MOF@P polymeric composite exhibited a sustained-release effect on HCPT, with a cumulative release rate of 30.18% in 72 h at pH 7.4. Furthermore, the cytotoxicity test demonstrated that the hydrophilic MOF and the MOF@P had low cell toxicities. The results indicate that the prepared MOF@P polymeric complex can be applied for the sustained release of HCPT in clinics.

## 1. Introduction

Hydroxycamptothecin (HCPT) is an effective antitumor drug extracted from Camptotheca acuminata in Sichuan Province, China [1]. It represents a new generation of anticancer drugs for treating numerous cancers, such as lung, gastric, prostate, and breast cancer. Eighteen camptothecin (CPT) alkaloids have been isolated from C. acuminata. Among them, 10-hydroxycamptothecin (10-HCPT) has the strongest antitumor activity, 30 times that of the other CPT alkaloids. The DNA topoisomerase activity can be inhibited by 10-HCPT, resulting in DNA single-stranded breaks. As a cell cycle-specific drug, HPCT has no cross-resistance with common antitumor drugs. It can delay the G2/M transition phase and mainly acts on S-phase cells [2,3,4,5]. The integrity of the six-membered hydroxylactone ring is the pharmacological activity core of HPCT, and hydroxyl modification enhances the HCPT stability and improves the therapeutic effect [6,7]. However, owing to the poor water solubility and adverse side effects of HCPT, its clinical application is limited [8,9]. Therefore, improving the water solubility and reducing the side effects of HCPT have become a research focus. Yang et al. [10] prepared 10-HCPT polymorphic nanoparticle dispersions via a supercritical antisolvent technology combined with high-pressure homogenization. The controllable size and large surface-to-volume ratio of the needle-shaped nanoparticles improve their dissolution characteristics, which enhanced their pharmacokinetic and pharmacokinetic properties. Ma et al. [11] used a modified emulsification–solvent evaporated method to successfully add hydroxycamptothecin, a water-insoluble antitumor agent, into lipid polymer hybrid nanoparticles (LPNs) formed from polylactic-co-glycolic acid (PLGA), 1,2-distearoyl-sn-glycero-3-phosphoethanolamine-N-(methoxy(polyethylene glycol)-2000) (DSPE-PEG2000), and lecithin. The optimized HCPT-loaded lipid polymer hybrid nanoparticles (HCPT-LPNs) were nano-sized, and the HCPT-LPNs were highly stable in plasma with sustained release characteristics related to pH and drug loading.

Metal–organic frameworks (MOFs) have been widely researched as porous materials in the past two decades [12]. MOFs can be used as nanocarriers because of their large specific surface areas and simple synthesis processes [13]. Currently, MOF materials with nanometer sizes have been widely used in medical imaging, disease diagnosis, and drug delivery [14], especially in the treatment of cancer [15]. In addition, Zwitterionic ligands have been used as organic linkers to synthesize multifunctional MOFs owing to their special charges and strong polarities, which can increase the hydrophilicity of MOFs [16,17,18,19]. Among these multifunctional MOFs, Chen’s team used amphoteric carboxylic acid ligands to coordinate with metal ions to synthesize a series of MOFs with water stability and water solubility [20,21,22,23,24]. Xie et al. [25] prepared three-dimensional (3D) and two-dimensional (2D) Ag-based zwitterionic MOFs and investigated their antimicrobial activities using a minimal inhibition concentration (MIC) test and killing kinetic assay. The two prepared Ag-based zwitterionic MOFs showed good water stability and solubility attributed to their aromatic ring natures and positively charged pyridinium of the ligands. Chen et al. [26] synthesized a luminescent water-stable terbium-based MOF ({[Tb(Cmdcp)(H_2_O)_3_]2(NO_3_)_2_·5H_2_O}n (1, H_3_CmdcpBr = N-carboxymethyl-(3,5-dicarboxyl)pyridinium bromide) used for the recyclable sensing of PO_4_^3−^ and Al^3+^ in tandem.

In some conditions, a single MOF cannot satisfy specific requirements well, and MOF nanocomposites are thus applied in biomedical fields, especially in drug delivery systems [27]. Lian et al. [28] prepared a tyrosinase-MOF nanoreactor and applied to activate the prodrug paracetamol in cancer cells in a long-lasting manner. The cytotoxicity of drug-resistant cancer cells arises from 4-acetamido-o-benzoquinone (AOBQ), the enzymatic conversion product of APAP, and from the subsequent generation of reactive oxygen species (ROS) and glutathione (GSH) depletion. Neisi et al. [29] prepared a nanocomposite with a higher porosity and conductivity using Cu-MOF and polypyrrole. The nanocomposites show a promising potential for different biomedical applications, such as biosensors and drug delivery. The liquid-solid solution (LSS) method was used to prepare nano-Fe-soc-MOF by Cai et al. [30], and they integrated the as-synthesized nanoscale Fe-soc-MOF with polypyrrole (PPy) to construct a multifunctional theranostic platform. The Fe-soc-MOF@PPy core–shell nanohybrids can be triggered by 808-nm laser irradiation to produce a photothermal effect for cancer therapy.

In order to improve the biocompatibility and water solubility of MOFs, water-soluble or hydrophilic chemicals are often used to compound or polymerize with MOFs. Methacrylic acid (MAA) [31] and acrylamide (AAm) [32] are usually polymerized with MOFs as monomers to augment their solubilities owing to the presence of carboxyl and amino groups, which easily form hydrogen bonds with certain atoms [33]. Ma et al. [34] described a novel water-compatible method appropriate for molecular imprinting, based on the use of a metal–organic gel (MOG) as the pore-forming agent. The MOG was synthesized from the combination of Fe (NO_3_)_3_ and benzene-1,3,5-tricarboxylic acid, which was used to prepare a molecularly imprinted polymer (MIP) containing imprints of levofloxacin using methacrylic acid as the functional monomer and ethylene glycol dimethacrylate (EGDMA) as the crosslinker. Cheng [35] developed a modified molecularly imprinted electrochemical sensor. The sensor was modified with BC/Cr_2_O_3_/Ag (BC/Cr_2_O_3_/Ag/MIP) and proposed for the sensitive and rapid detection of nitrofurazone (NFZ). MIP was prepared by precipitation polymerization using acrylamide and α-methacrylic acid as a bifunctional monomer and NFZ as a template.

**Scheme 1:**
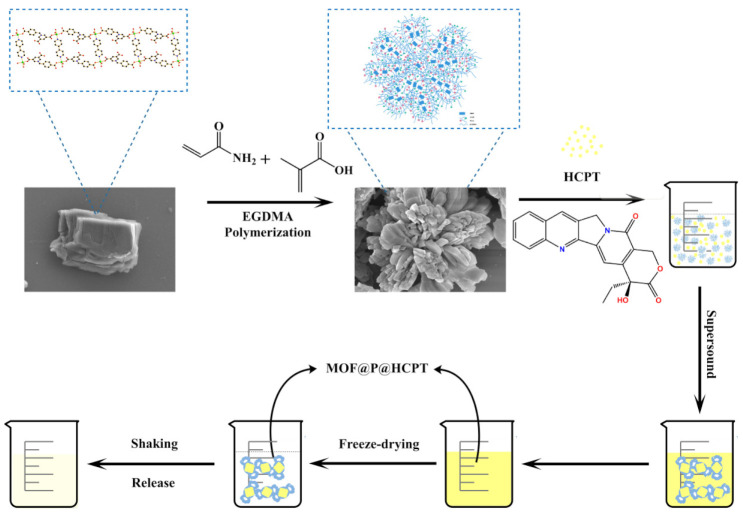
Schematic diagram of the preparation and drug delivery of HCPT.

Although some nanomaterials that can improve the solubility of HCPT in water and have a slow release effect have been reported in the above-mentioned references, these materials are essentially insoluble in water and have no obvious solubilization effect on HCPT. Therefore, in this study, a hydrophilic MOF was prepared using zwitterionic ligands as organic linkers. Then, MAA and AAm were employed as monomers to polymerize with the synthesized hydrophilic MOFs to obtain water-soluble polyacid polymer composites (MOF@P). The polymer MOF@P was tested to have a solubility of 370 μg/mL in pure water and was applied to increase the solubility of hydrophobic compounds and for HCPT delivery (Scheme 1). The results showed that the solubility of HCPT was comparatively improved using the polymeric material. Furthermore, MOF@P had a sustainable delivery of HCPT, and the cumulative release rate of HCPT in vitro was about 30% within 72 h at a pH 7.4 buffer solution. The cytotoxicity studies also indicated that the hydrophilic MOF and the composite material had good biocompatibility, which suggests their potential use as drug carriers in cancer treatment.

## 2. Materials and Methods

### 2.1. Reagents and Apparatus

Reagents, including 4-bromomethyl benzoic acid, 3,5-pyridinedicarboxylic acid, cupric acetate monohydrate (CuAc_2_·H_2_O), phosphate, 4,4-bipyridine, methacrylic acid (MAA), acrylamide (AAm), ethylene glycol dimethacrylate (EGDMA), 2,2′-Azobis(2-methylpropionitrile) (AIBN), HCPT, CPT, and paclitaxel (PTX), were purchased from Aladdin Biochemical Technology Co., Ltd. (Shanghai, China). N, N-Dimethylformamide (DMF), methanol, ethanol, ethyl ether, and acetate were purchased from Tianjin Deen Chemical Reagent Co., Ltd. (Tianjin, China). The reagents used in the cell experiments were purchased from Zhengzhou Purcell Life Technology Co., Ltd. (Zhengzhou, China). The HL-7702 and HepG2 cell lines were purchased from the cell bank of the Chinese Academy of Sciences (Shanghai, China). The other chemicals used in this work were of analytical grade.

Elemental analyses of C, H, and N were conducted on a PerkinElmer 24000-II elemental analyzer. The contact angles of the materials were measured by a liquid–solid interface analyzer (DM300, Kyowa Interface Science Co. Ltd., Niiza, Japan). X-ray diffraction (XRD) patterns were recorded on a Bruker D8 Advance diffractometer (Bruker, Karlsruhe, Germany) by using Cu Kα radiation from 5° to 60° (2θ). A JSM-7610F scanning electron microscope (SEM) (JOEL, Peabody, MA, USA) was used to characterize the shapes of the nanomaterials. A nanometer particle size potential analyzer was applied to test the particle size and Zata potential (Malvern Zetasizer Nano ZS90, Malvern Ltd., Malvern, UK). A Bruker Vertex 70 Fourier-transform infrared (FTIR) spectrometer (Bruker Corporation, Karlsruhe, Germany) was employed to obtain the FTIR spectra. A TU-1900 spectrophotometer (Beijing, China) was to measure the UV–Vis absorption spectrum. A (3-[4,5-dimethylthiazol-2-yl]-2,5 diphenyl tetrazolium bromide) (MTT) assay was conducted on a microplate reader (CLARIO star, BMG LABTECH, Ortenberg, Germany).

### 2.2. Preparation of Hydrophilic MOF

The synthesis of H_3_CbdcpBr (N-(4-carboxybenzyl))-(3,5-dicarboxyl) pyridinium bromide) procedure was followed as reported with some modifications [36]. First, 4.30 g of 4-bromomethyl benzoic acid and 3.34 g of pyridine 3,5-dicarboxylate were dissolved in 10 mL and 20 mL of DMF solution, respectively, and they were uniformly mixed. The white precipitate was formed in the reaction at 60 °C for 24 h. The precipitate formed was washed with a mixture of methanol and ether (V:V = 1:1) and then dried in a vacuum drying oven. The structure of the product was confirmed by the FTIR and ^1^Hydrogen-1 nuclear magnetic resonance (^1^H-NMR) analyses. The FTIR data were as follows: FTIR (cm^−1^) ν: 3426(m), 3036(s), 1728(s), 1642(s), 1425(s), 1306(s), 1220(s), 1166(s), 1103(s), 821(m), 749(s), 681(s), 626(s), 586(s), 509(s), and 432(s). The ^1^H-NMR data were as follows: δ(300 MHz, D2O): 9.29(s, 2H), 9.16(s, 1H), 7.94(d, J = 8.2Hz, 2H), 7.55(d, J = 8.3Hz, 2H), and 5.97(s, 2H). The FTIR and ^1^H-NMR data proved that H_3_CbdcpBr was successfully synthesized.

Then, 38.2 mg of H_3_CbdcpBr was dissolved in 20 mL of double-distilled water, and the pH was adjusted from 6.0 to 6.5 with 0.2 M of NaOH. The aqueous solution of 1.5 mL of CuAc_2_·H_2_O (20 mg) was added and stirred at room temperature for 30 min, and 1 mL of 4,4′-bipyridine (15.6 mg) dissolved in DMF was slowly added and stirred at a reduced rotational speed for 5 min. The blue filtrate was obtained after filtration. After one week, the crystals in the filtrate were washed with ether and dried in a vacuum. The hydrophilic MOF materials were obtained after this process.

### 2.3. X-ray Crystallographic Study

The MOF crystal with a size of 0.17 × 0.14 × 0.06 mm^3^ was selected for the structure determination. The samples were placed in a Bruker D8 venture photo II CCD diffractometer (Bruker, Germany), and the scanning ranges were −8 ≤ h ≤ 8, −14 ≤ k ≤ 14, and −16 ≤ l ≤ 16. The structure of the compound was analyzed by the shell XT program, which was refined by the least square method F^2^ with the shell XL package, and the diffraction intensity was corrected by the Lp factor and empirical absorption. A summary of the key crystallographic information is shown in Table 1.

### 2.4. Synthesis of Water-Soluble Composites (MOF@P)

First, 20 mg of hydrophilic MOF with some proportion of AAm and MAA were mixed together with 6 mL of ethanol. The mixture was stirred at room temperature for 60 min with the addition of some quantities of ethylene glycol dimethacrylate (EGDMA) and azobisisobutyronitrile (10 mg) dissolved in 4 mL of ethanol. The reaction of this mixture was carried out at 65 °C for 20 h after ultrasonic treatment for 30 min. The product resulted from this reaction was washed with methanol–acetic acid (*v*:*v* = 9:1) and ethanol successively and then dried in a vacuum oven to obtain water-soluble composites. The solubility of the composite in pure water at 20 °C was determined to be 370 μg/mL by UV spectrophotometry.

### 2.5. Drug Loading on Water-Soluble Composites (MOF@P)

The calculation of the drug loading content was carried out according to the method developed by our research group [37]. Separate amounts of the composite material (1 mg) and hydroxycamptothecin (1 mg) were added to 3 mL of methanol and ultrasonicated for 2 h. Then, 10 mL of distilled water was slowly added to the mixture and ultrasonicated for 40 min to remove the volatile organic reagent. After the complete removal of the volatile organic reagent, the solution was centrifuged at 8000 rpm for 10 min; the supernatant was filtered through a 0.45-μm membrane and was sealed away from light and stored at 4 °C. The drug-loading content (DLC) and efficiency (DLE) of the material were determined by detecting the drug concentration in the supernatant using the ultraviolet spectrophotometry method. The following equations were used to calculate the DLC (Equation (1)) and DLE (Equation (2)) of the nanocarrier:

(1) DLC (μg/mg) = amount of drug in materials/amount of materials

(2) DLE (%) = amount of drug in materials/amount of drug added × 100%

### 2.6. Drug Release In Vitro under pH-Controlled Conditions

After the above-obtained supernatant was freeze-dried, 1 mg of powder was accurately weighed, dissolved in 5 mL of phosphate-buffered saline (PBS) buffer solution, and then transferred into a dialysis bag. The bag was placed into a 100-mL PBS buffer solution with a pH = 6.5 or pH = 7.4, and the solution was shaken in the dark at 37 °C. At a certain time, a 3 mL sample was removed, and its absorbance value was measured, and the same volume of corresponding fresh PBS solution was added at the same time. The following equation was used to calculate the cumulative release efficiency:

(3) $$\mathrm{Cumulative}\;\mathrm{release}\;(\%)=\frac{C_{n}\times V_{0}+V_{i}\sum_{i=1}^{n-1}C_{i}}{m}\times 100,$$

where *C_n_* (µg/mL) is the HCPT concentration sampled at a specific time. *V_i_* and *V*_0_ (mL) refer to the sampling volume and the total volume of the release medium, respectively, and *m* (mg) represents the mass of materials.

### 2.7. Cytotoxicity Assay

The human hepatocellular carcinoma cell line (HepG2) and human normal liver cell line (HL-7702) was cultured in Dulbecco’s modified Eagle’s medium (DMEM) supplemented with 10% (*v/v*) fetal bovine serum (FBS) and RPMI1640 with 20% FBS, respectively, in a humidified atmosphere containing 5% CO_2_ at 37 °C.

The cytotoxicity of all the materials was evaluated via an MTT assay using HepG2 and HL-7702 cells, which were cultured in a 5% CO_2_ cell incubator at 37 °C. The cells in the logarithmic growth phase were seeded into 96-well plates, with 3 × 10^3^ cells per well. Then, they were incubated at different concentrations of hydrophilic MOF, MOF@P, and MOF@P@HCPT for 24, 48, and 72 h, respectively. The control group was treated with a normal medium. Finally, the absorbance of each well was measured at 492 nm in triplicate.

## 3. Results

### 3.1. Characterization of the Synthesized Materials

**Figure 1:**
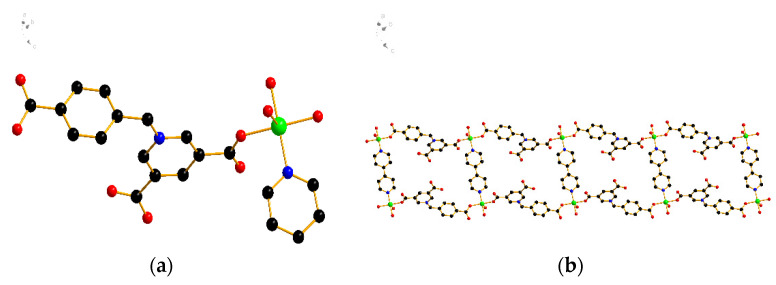
Crystal structure of the prepared MOF. (**a**) The asymmetric element of MOF. (**b**) The one-dimensional grid plane structure of MOF. Atomic colors: Cu (green), N (blue), O (red), and C (black).

The single-crystal X-ray diffraction analysis demonstrated that the hydrophilic MOF belonged to the P-1 space group of the triclinic system, and its asymmetric unit contained one [Cu(Cbdcp)(bipy)_1/2_] molecule and two dissociative water molecules. The two carboxyl groups of the ligand Cbdcp were connected with Cu^2+^, and the Cu^2+^ was connected with bipy (Figure 1a,b). The bond length and bond angle of the MOF are shown in Table 2.

**Figure 2:**
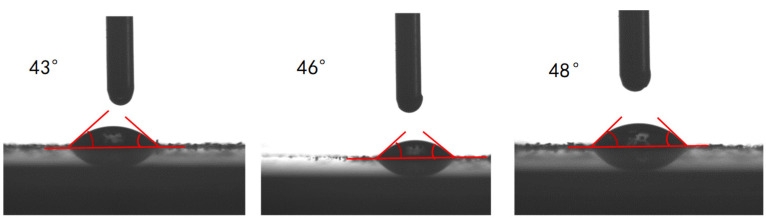
Static water contact angle results for the MOF.

The contact angle is one of the main parameters for quantifying the wettability of a liquid to a material surface. Figure 2 illustrates the changes due to the droplet imbibition into the materials in the three tests. Contact angles less than 90° and 50° indicated a hydrophilic solid [38]. The contacts angle of the MOF synthesized in this work was 46°, which means that the MOF was hydrophilic.

**Figure 3:**
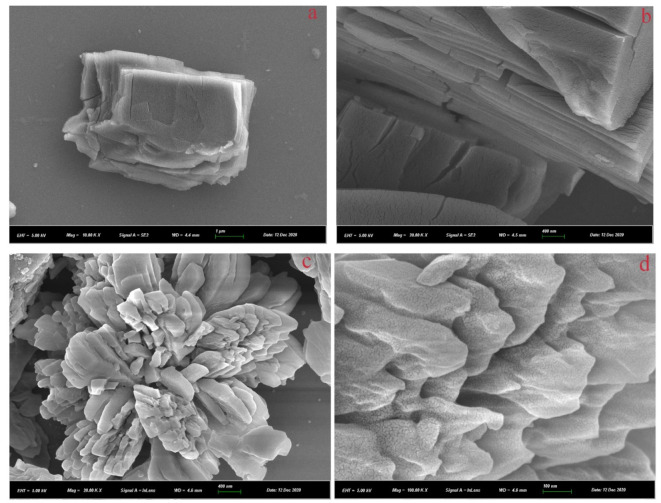
SEM images of the MOF (**a**,**b**) and MOF@P (**c**,**d**).

The morphologies of the hydrophilic MOF and MOF@P were observed by scanning electron microscopy (SEM). The MOF structure was observed to be stacked layer by layer (Figure 3a,b). The structure of the material changed from lamellar to a flower-like structure after compounding (Figure 3c). This change can be attributed to the polymerization of an MOF with a monomer and crosslinker, which is formed from connecting multiple blocks of MOFs.

**Figure 4:**
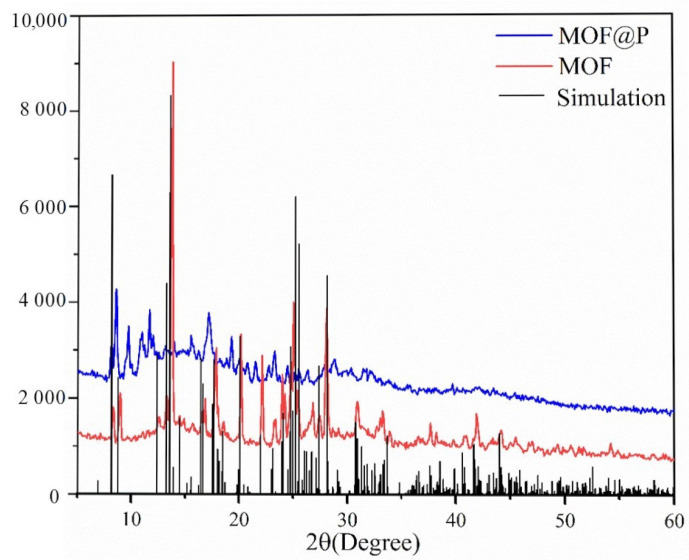
XRD patterns of the simulated MOF, prepared MOF, and MOF@P.

The phase purity and crystal structure of the materials were verified using PXRD patterns (Figure 4). The PXRD pattern of the MOF was consistent with the diffraction data of a single crystal, which indicates that the material was highly crystalline. It can be clearly seen that the peak intensities of MOF@P decreased dramatically because of the influence of the amorphous shell, which was mainly due to the successful coating procedure [39].

**Figure 5:**
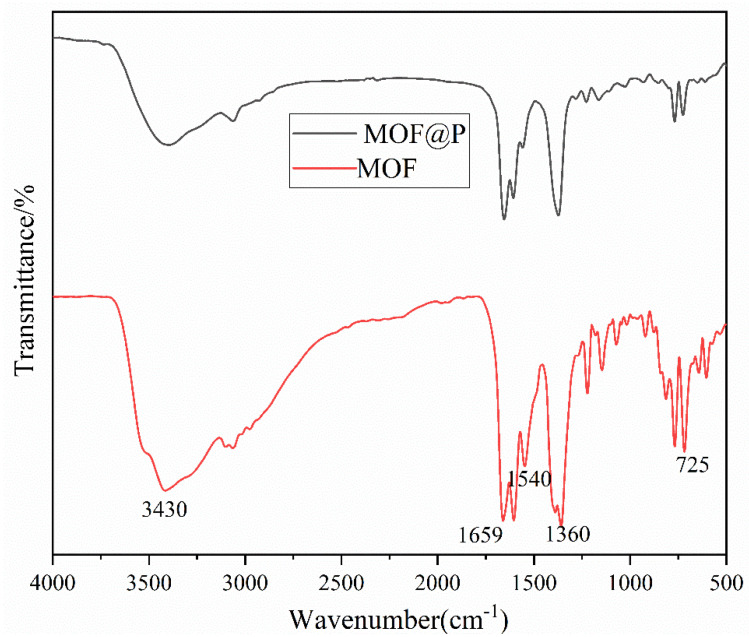
FTIR spectra of the MOF and MOF@P.

The FTIR spectra of the synthetic materials were evaluated to confirm that the expected synthetic reaction of the MOF and the MOF@P occurred. The results of the FTIR spectra showed that the wavelength of 3430 cm^−1^ was the stretching vibration peak of -OH (Figure 5). The wavelengths of 1659 and 1360 cm^−1^ were the asymmetric stretching vibration peaks of C=O, which belonged to the symmetric stretching and antisymmetric stretching of -COOH. In addition, the 1540 cm^−1^ wavelength was the C=C absorption peak of a benzene ring on the MOF. The strong characteristic peak at 725 cm^−1^ was the stretching vibration peak of Cu-O [40], which confirmed the polymerization of the participating MOF.

**Figure 6:**
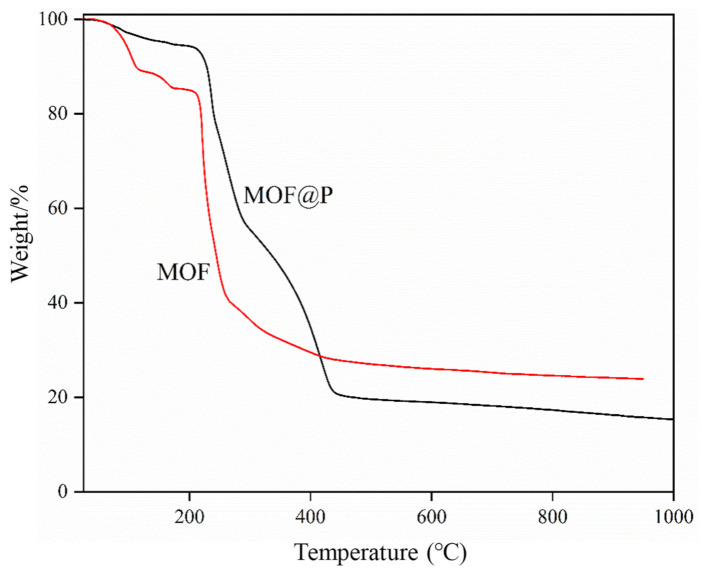
TGA curves of the MOF and MOF@P.

Pyrolysis experiments were conducted using the thermogravimetric method to investigate the thermal stabilities of the MOF and MOF@P under nitrogen atmospheres. Two consecutive weight loss stages were observed on the TGA curve of the MOF (Figure 6). In the first stage, a 14.23% (calculated 14.09%) weight loss occurred at the temperature range of 25–175 °C, and this loss was assigned to the disappearance of two crystal water molecules and two coordinated polymer water molecules. However, in the second stage, a 27.39% (calculated 27.47%) weight loss occurred at the temperature range of 175–1000 °C, and this weight loss was assigned to the loss of organic ligands [41]. Thus, the structural formula of the hydrophilic MOF can be defined as [Cu(Cbdcp)(bipy)_1/2_(H_2_O)_2_]·2H_2_O. Moreover, after 200 °C, MOF@P starts to lose weight. These results proved that the temperature could affect the thermal stability in the field of drug loading.

### 3.2. Optimization of the MOF@P synthesis Parameters

Although we have synthesized the hydrophilic MOF, its water solubility was not ideal due to its spatial structure. Therefore, it is necessary to modify its surface to obtain certain water-soluble materials for drug loading. MAA and AAm were adopted as functional monomers to polymerize with the hydrophilic MOF to form a water-soluble complex. The function of the crosslinking agent is to fix the functional groups of functional monomers to form a stable rigid polymer. The preparation of MOF@P with a varied ratio of EGDMA was conducted at a fixed ratio of MAA:AAm (molar ratio 1:1), and 20 mg of MOF was used to investigate the crosslinker effect on drug loading. It was observed that the increase in the content of EGDMA led to a floating up and down of the HCPT loading efficiency (Table 3). When the amount of EGDMA approached 1 mmol, the drug loading of HCPT reached a maximum concentration of 237.8 μg/mg. It was observed that the drug loading rate decreased as the crosslinker content increased. Therefore, 1 mmol of EGDMA was used as the optimal amount to participate in the polymerization.

The drug loading capacity of the polymers of MOF@P was influenced by different molar ratios of functional monomers. The influenced drug loading effect for the MOF@P was investigated by varying the MAA/AAm ratio and keeping the EGDMA at 1 mmol. The drug loading rate first increased and then decreased with the change of the MAA/AAm ratio (Table 4). It was observed that the drug loading reached its peak when the molar ratio was 1:1. In summary, the optimization of MOF@P synthesis was achieved when the EGDMA and monomer ratios were 1 mmol and 1:1, respectively.

### 3.3. Drug Solubilization Research

**Figure 7:**
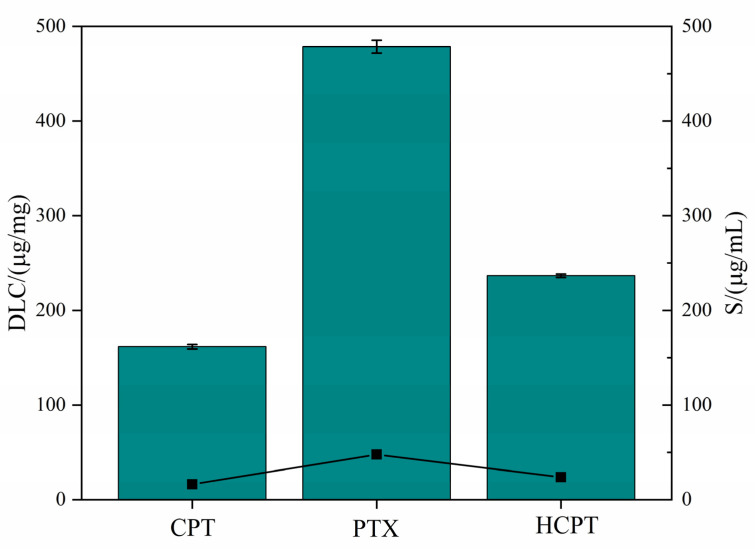
Drug loading and solubility of MOF@P for HCPT, CPT, and PTX.

The solubility of the above obtained polymeric composite in water was examined at room temperature (20 °C) by a UV spectrophotometer. It was examined to be 370 μg/mL. This result was applied to investigate the solubilization effect for three different water-insoluble drugs. Camptothecin (CPT) and paclitaxel (PTX), which have similar water-insolubilities as HPCT, were employed to investigate the water solubility effect of MOF@P. Figure 7 shows that the solubility of HCPT in the MOF@P aqueous solution was 29 times higher than that of the solubility of HCPT in water (2.187 μg/mL), which indicates that the polymer has a noticeable solubilization effect on the HCPT [42]. In addition, the solubilities of CPT and PTX in the MOF@P aqueous solution were six times [43] and 41 times [44] those in water. Therefore, the polymers can be used as carriers to increase the water solubility of insoluble drugs.

### 3.4. Stability of Drug-Loaded MOF@P

**Figure 8:**
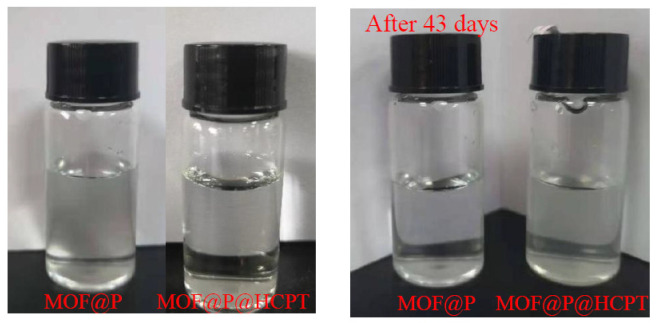
Photos of blank MOF@P and MOF@P@HCPT.

The aqueous solution of the drug-loaded polymer prepared by the optimal formula was clear and light-transparent yellow, while the aqueous solution of the blank polymer was colorless and transparent (Figure 8). When the drug-loaded polymer was stored in the refrigerator at 4 °C for some time, there was no change observed. After 43 days of observation, the solution was still clear. These observed results indicated that the drug-loaded polymer was comparably stable.

Using water as the solvent, the zeta potentials and average particle sizes of MOF, MOF@P, and MOF@P@HCPT (0.10 mg/mL) were tested. The results are shown in Table 5. Due to a slightly positively charged MOF (0.31 mV), therefore, the negative charge of MOF@P mainly comes from the polymer. While the polymer monomers contain carbonyl and carboxyl groups, during the formation of polymer composites, the hydrophilic chains of MAA and AAm surrounded the MOF core, neutralizing and shielding some charges of MOF; thus, the Zeta potential of the prepared MOF changed to −0.70 mV when polymerized with MAA and AAm. It was reported that the surface charges of nanopolymers can affect their distribution in vivo. The less negatively charged and swallowed the nanopolymer is, the longer its circulation time in the blood will be [45,46]. The zeta potential is an important index for measuring the polymer stability. A higher zeta potential absolute value indicates more stable polymers [37]. The zeta potentials of MOF@P were more negative after HCPT was loaded onto the MOF@P, and it was changed to −4.58 mV. This change was attributed to the increase in the size of the nanocomposite particles, which can be verified by the average particle sizes of MOF@P and MOF@P@HCPT (~68.14 nm for MOF@P, with a polymer dispersion index of 0.163, and 278.29 nm for MOF@P@HCPT with a polymer dispersion index of 0.144). According to References [47,48,49], drug-loaded polymers prepared by the physical embedding method solubilize drugs in polymers mainly through hydrophobic force and a hydrogen bond. The experimental results showed that the average particle size of MOF@P@HCPT was larger than that of MOF@P, which may be due to the fact that the HCPT molecules entered the polymer core and occupied part of the space, which led to the outward expansion of the polymer core. In addition, there was a hydration layer in the polymer, so MAA and AAm could be closely combined before the HCPT was loaded. When the drug entered the polymer core, the core size became larger, and the combination of the polymer chains became loose, which made the hydration layer thicker and led to an increase of the polymer size. Owing to the increase in particle size, the density of the MAA/AAm on the MOF surface decreased. This change in density resulted in less MAA and AAm needed to shield the MOF molecules, so that the composites exhibited a lower zeta potential, and thus, the drug-loaded polymers had higher stability.

Furthermore, we measured the zeta potentials of MOF@P and MOF@P@HCPT after 45 days of storage at 4 °C. Owing to the increase in the number of negative charges, the zeta potential of MOF@P changed from −0.70 mV to −2.33 mV, while the zeta potential of MOF@P@HCPT decreased from −4.58 mV to −4.97 mV. These results demonstrate that the nanocomposite polymers and their drug-loaded systems can be stable for a long time.

### 3.5. In Vitro Release

**Figure 9:**
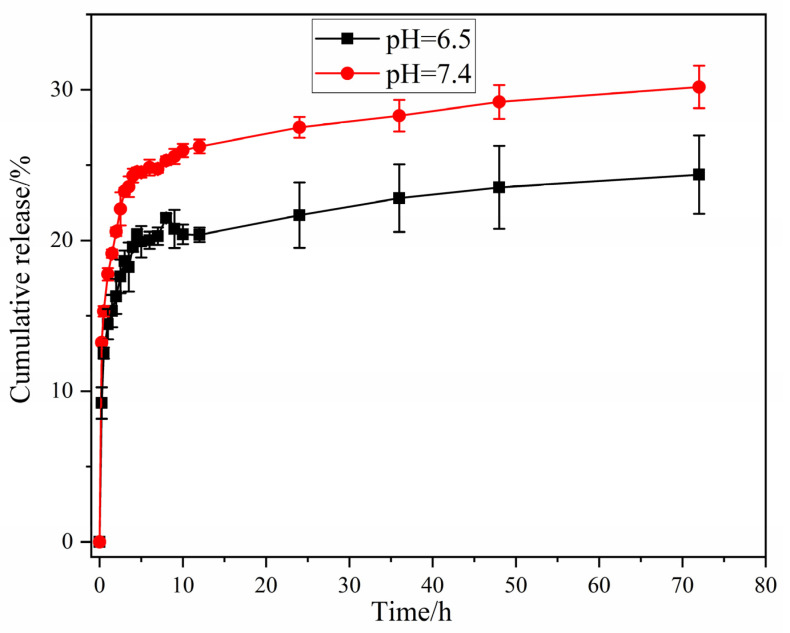
Cumulative release ratio of the MOF@P after drug loading in the buffer.

A sustained drug release is an important index of nanodrug delivery systems. The in vitro drug release behavior of HCPT in the MOF@P in PBS (pH 7.4 and 6.5) was assessed (Figure 9). The HCPT release rate in the pH 7.4 PBS buffer was higher than that in the pH 6.5 PBS buffer, and the cumulative release time exceeded 72 h. Under a pH 7.4 buffer, the cumulative release rate of the composite was approximately 30% within 72 h, whereas the cumulative release rate was approximately 20% at 72 h in the pH 6.5 buffer. Moreover, the HCPT release was much faster at a pH of 7.4 than that at a pH of 6.5 within 72 h. This was because the higher the solution pH, the higher the HCPT solubility, as the HCPT was converted into a relatively higher water-soluble carboxylate form, which was conducive to faster releases [50].

Generally, the Korsmeyer–Peppas [51] and Sigmoidal models [52] are applied to describe the drug release kinetics of the composite materials, and the equations are shown as follows:

(4) $$Q=Kt^{n}$$

(5) $$Q=\frac{R_{s}}{1+e^{-k_{s}(t-t_{50})}}$$

where *Q* represents the cumulative release percentage at time *t* (*h*), *K* is the kinetic constant, and *n* is the diffusion exponent. The theoretical maximum release rate is *R_s_* (%), and *k_s_* and *t*_50_ are the release kinetic constants.

The results of the kinetic parameters for the two model are summed up in Table 6. It can be seen that, at pH 7.4, the experimental data conformed well to the Korsmeyer–Peppas model, and according to the fitting results of Korsmeyer–Peppas in the MOF@P release system in Table 6, n was 0.13 at pH 7.4 which was less than 0.45, revealing that the release kinetics process of the system conformed to the Fick diffusion mechanism [51]. In other words, the interaction between MOF@P and the drug was relatively weak, and the concentration gradient of HCPT inside and outside the dialysis bag was larger at the initial stage, leading to a relatively faster release at the first few hours. After a certain number of hours, the concentration gradient became lower, and HCPT had poor solubility in the PBS buffer, leading to a slower release in the following hours. In addition, the K value at pH = 7.4 was higher than that at pH = 6.5, and the higher K value indicated that the drug release rate was faster, which proved that the drug could be released faster in the buffer solution at pH = 7.4 than in the buffer solution at pH = 6.5, which was consistent with the results in Figure 9 [52]. However, for the pH 6.5 release system, the experimental data was neither consistent with the Korsmeyer–Peppas model nor well-fitted to the Sigmogenic model. The abnormal non-Fick spread was described by the Sigmoidal release function. The changes in drug diffusion and internal stresses resulted in abnormalities. The relaxation and diffusion macromolecular time scales were similar in anomalous non-Fick diffusion [53,54,55,56,57].

### 3.6. Cytotoxicity Assay

**Figure 10:**
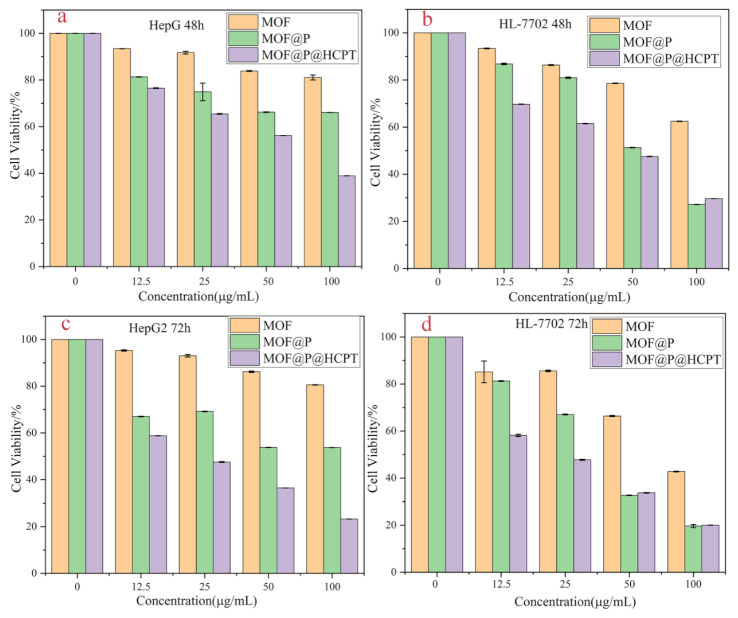
Cell viability of HepG2 cells and HL-7702 cells treated with MOF, MOF@P, and MOF@P@HCPT in different concentrations at 48 h (**a**,**b**) and 72 h (**c**,**d**).

The effects of the hydrophilic MOF, MOF@P, and MOF@P@HCPT on the cell survival rate of HepG2 and HL-7702 were detected through the MTT method to evaluate the biocompatibility of the synthetic materials. The inhibition of these three materials on the two kinds of cells were all time concentration-dependent (Figure 10). The inhibitory effect of MOF@P@HCPT on HepG2 cells was higher than that of MOF and MOF@P after 48 h of treatment, and a possible reason for the toxicity perhaps resulted from the released anticancer drug HCPT. However, when the concentrations of MOF and MOF@P were up to 25 μg/mL, they showed low cytotoxicity with less than a 20% cell death rate, which means that these materials have good biocompatibility. In addition, at the same concentration of these three materials, the inhibition of HepG2 cell viability was higher after being treated for 72 h than for 48 h. The IC_50_ of these materials toward cells were calculated to evaluate the cytotoxicity. For HL-7702 cells, the IC_50_ of MOF@P and MOF@P@HCPT at 48 h were 64.66 μg/mL and 56.49 μg/mL, respectively. After 72 h, the IC_50_ were 48.28 μg/mL and 38.09 μg/mL, respectively. It can be seen that the inhibitory effects of MOF@P and MOF@P@HCPT on HL-7702 cells increased with time, and the cytotoxicity of MOF@P@HCPT was significantly higher than that of MOF@P without the drug materials. This may be due to the release of HCPT in MOF@P. Additionally, for HepG2 cells, the repress effect was also time-dependent. The IC_50_ of MOF@P and MOF@P@HCPT at 48 h were 124.28 μg/mL and 70.72 μg/mL, respectively. After 72 h, the IC_50_ were 88.68 μg/mL and 40.81 μg/mL, respectively. It can be seen that the inhibitory effect of the materials on HepG2 cells was relatively lower than that on HL-7702 cells. However, the repress effect was comparable to MOF@P@HCPT after 72 h. Therefore, it can be concluded from the results that MOF@P@HCPT had a sustained-release effect on HCPT. Furthermore, the MOF and MOF@P had low cytotoxicity, indicating their potential use as drug carriers. Our following work will be to improve the material properties to make them targeted to sustainably kill cancer cells.

## 4. Conclusions

A hydrophilic MOF was synthesized and polymerized with methacrylic acid and acrylamide using EGDMA to form a water-soluble polyacid polymer (MOF@P) with a solubility of 370 μg/mL. The results showed that the MOF@P had a specific solubilization effect on HCPT, and the drug loading of HCPT reached a maximum concentration of 237.8 μg/mg. Moreover, the composite exhibited a sustained-release effect on HCPT, and the cumulative release rates of HCPT in 72 h were 30.18% and 22.40% at pH 7.4 and 6.5, respectively. The cytotoxicity test showed that the hydrophilic MOF and the MOF@P had low cell toxicity. Therefore, the water-soluble polyacid polymer featured good solubilization, sustained-release properties, and good biocompatibility; thus, it has application potential as a carrier in drug solubilization and sustained-release drug delivery.

## Figures and Tables

**Table 1:** Crystallographic data for the prepared MOF.

The Key Crystallographic Information of MOF	
Empirical formula	C_20_H_19_CuN_2_O_10_
Formula weight	510.91
Temperature/K	296
Crystal system	triclinic
Space group	P-1
a/Å	7.2164 (7)
b/Å	11.6530 (11)
c/Å	13.4323 (11)
α/°	108.287 (3)
β/°	93.184 (3)
γ/°	102.297 (4)
Volume/Å^3^	1038.75 (17)
Z	2
ρcalcg/cm^3^	1.633
μ/mm^−1^	1.114
F (000)	524.0
Crystal size/mm^3^	0.17 × 0.14 × 0.06

**Table 2:** Main bond lengths (Å) and angles (°) for MOF.

**Bond Lengths (Å)**			
Cu1-O1	1.9365 (16)	Cu1-O2W	2.2092 (18)
Cu1-O1W	1.9869 (16)	Cu1-N2	2.028 (2)
Cu1-O5^1^	1.9654 (17)		
**Bond Angles (°)**			
O1-Cu1-O1W	85.77 (7)	O1W-Cu1-N2	172.49 (7)
O1-Cu1-O5^1^	172.24 (7)	O5^1^-Cu1-O1W	91.04 (7)
O1-Cu1-O2W	87.34 (7)	O5^1^-Cu1-O2W	99.91 (8)
O1-Cu1-N2	93.34 (8)	O5^1^-Cu1-N2	88.91 (7)
O1W-Cu1-O2W	93.12 (7)	N2-Cu1-O2W	94.28 (7)

**Table 3:** Effects of the crosslinking agent contents on drug loading content and efficiency for MOF@P.

nEGDMA (mmol)	DLE (%)	DLC (μg/mg)
0.50	14.2	141.5 (2.13) ^a^
0.75	23.1	231.4 (0.73)
1.00	23.8	237.8 (0.50)
1.25	21.4	214.0 (0.82)
1.50	16.4	164.3 (0.64)

^a^ The data in the parentheses are the standard deviations.

**Table 4:** Effects of the monomer molar ratios on the drug loading content and efficiency.

nMAA:nAAm	DLE (%)	DLC (μg/mg)
1:4	17.6	176.0 (5.52) ^a^
2:3	23.5	235.3 (1.95)
1:1	23.8	237.8 (1.05)
3:2	20.3	202.5 (2.86)
4:1	18.5	184.6 (4.56)

^a^ The data in the parentheses are the standard deviations.

**Table 5:** Zeta potential and average particle sizes of the materials.

	Sample	MOF	MOF@P	MOF@P@HCPT
Parameter				
Zeta potential (mV)		0.31 (0.21) ^a^	−0.70 (0.024)	−4.58 (0.15)
Size (nm)/PDI		1285.7/0.192 (62.57)	68.14/0.163 (9.00)	278.29/0.144 (17.76)

^a^ The data in the parentheses are the standard deviations.

**Table 6:** Correlation coefficient (R^2^) of the release mechanism model Sigmoidal and Korsmeyer–Peppas according to various pHs.

Condition	Korsmeyer–Peppas			Sigmoidal Model			
	K	n	R^2^	k_s_	R_s_	t_50_	R^2^
pH = 6.5	14.29	0.14	0.75	1.16	21.03	0.716	0.85
pH = 7.4	18.47	0.13	0.90	1.17	26.10	0.666	0.84
